# Anthelmintic effect of carob pods and sainfoin hay when fed to lambs after experimental trickle infections with *Haemonchus contortus* and *Trichostrongylus colubriformis*[Fn FN1]

**DOI:** 10.1051/parasite/2014074

**Published:** 2014-12-22

**Authors:** Celia Arroyo-Lopez, Foteini Manolaraki, Anastasios Saratsis, Katerina Saratsi, Alexandros Stefanakis, Vasileios Skampardonis, Nikolaos Voutzourakis, Hervé Hoste, Smaragda Sotiraki

**Affiliations:** 1 Veterinary Research Institute – Hellenic Agricultural Organization Demeter 57001 Thermi, Thessaloniki Greece; 2 UMR 1225 IHAP INRA/ENVT, École Nationale Vétérinaire de Toulouse 23 Chemin des Capelles 31076 Toulouse Cedex France

**Keywords:** Gastrointestinal nematodes, Carob (*Ceratonia siliqua*), Sainfoin (*Onobrychis viciifolia*), Tannin, Polyphenol, Nutraceuticals

## Abstract

The aim of the study was to compare the *in vivo* anthelmintic activity of sainfoin hay (*Onobrychis viciifolia*) and carob pod meal (*Ceratonia siliqua*) against gastrointestinal nematodes. Seven days before infection, 64 naive lambs were assigned to four different groups: Group S received sainfoin hay and group CAR was fed with carob pods. The remaining lambs received lucerne hay (*Medicago sativa*) and were assigned to positive (non-treated, NT) and negative (treated, T) control groups (treatment with albendazole). On day 0, lambs were artificially trickle infected for 6 weeks, with a mixture of infective larvae of *Haemonchus contortus* and *Trichostrongylus colubriformis.* Parasitological and pathophysiological parameters were measured repeatedly during the 2-month study. Compared to the NT group, decreases in egg excretion were observed in the CAR and S groups with significant differences only found for sainfoin (*p* < 0.05). At necropsy, group S showed decreases in the total worm numbers of both nematode species with significant differences for *H. contortus*. In contrast, no differences were noticed for the CAR group. Compared to the NT group, lower values for fecundity of female *H. contortus* were found in the S and CAR groups, however differences were non-significant. No differences in body weight gains were found between groups. Consistent results were found showing significantly higher packed cell volume (PCV) values in the T and S groups compared to NT and CAR groups. Overall, these results confirm a positive effect associated with the feeding of lambs with tanniniferous resources on host resilience (PCV values) and against gastrointestinal parasitic nematodes by affecting some biological traits of worm populations (e.g. eggs per gram of faeces and worm numbers). However, the anthelmintic effects differed between the two tannin-containing resources, which might be associated with the quantity and/or quality of secondary metabolites (condensed tannins and/or other polyphenols).

## Abstract (fr)

L’objectif de l’étude était de comparer l’activité antihelminthique *in vivo* du foin de sainfoin (*Onobrychis viciifolia*) et des gousses de caroubier (*Ceratonia siliqua*) contre les nématodes gastro-intestinaux. Sept jours avant l’infection, 64 agneaux naïfs ont été répartis en quatre groupes : le groupe S a reçu du foin de sainfoin et le groupe CAR a été nourri avec des gousses de caroube. Les agneaux restants ont reçu du foin de luzerne (*Medicago sativa*) et ont été répartis en des groupes de contrôle, un groupe positif (non traité, NT) et un groupe négatif (traité, T) (traitement par l’albendazole). Au jour 0, les agneaux ont été artificiellement infectés pendant six semaines, avec un mélange de larves infectantes d’*Haemonchus contortus* et *Trichostrongylus colubriformis*. Les paramètres parasitologiques et physiopathologiques ont été mesurés à plusieurs reprises pendant les deux mois de l’étude. Par rapport au groupe NT, une diminution de l’excrétion des œufs a été observée dans les groupes CAR et S, avec des différences significatives seulement pour le sainfoin (*p* < 0.05). À l’autopsie, le groupe S a montré une diminution du nombre total de vers de deux espèces de nématodes, avec des différences significatives pour *H. contortus*. En revanche, aucune différence n’a été remarquée pour le groupe CAR. Par rapport au groupe NT, des valeurs inférieures de fécondité des *H. contortus* femelles ont été trouvées dans les groupes S et CAR mais les différences n’étaient pas significatives. Aucune différence dans les gains de poids de corps n’a été trouvée entre les groupes. Des résultats cohérents ont été trouvés concernant les valeurs de l’hématocrite, significativement plus élevées dans les groupes T et S par rapport aux groupes NT et CAR. Dans l’ensemble, ces résultats confirment un effet positif lié à l’alimentation des agneaux avec des ressources à tannins, sur la résilience de l’hôte (valeurs d’hématocrite) et contre les nématodes gastro-intestinaux, en affectant certains traits biologiques des populations de vers (par exemple, œufs par gramme de fèces et nombres de vers). Toutefois, les effets antihelminthiques différaient entre les deux ressources contenant des tannins, ce qui pourrait être associé à la quantité et / ou à la qualité de métabolites secondaires (tannins condensés et / ou autres polyphénols).

## 1. Introduction

Gastrointestinal parasitic nematodes (GINs) strongly affect livestock health, welfare and production in small ruminants because of the major economic losses, clinical signs and possible deaths which they provoke. Until now, the main mode of control of these parasitic diseases relied on chemical anthelmintics (AHs). However, the prevalence of AH resistance within worm populations is now a worldwide phenomenon [15]. In addition, the societal demand to reduce chemicals in agriculture in order to avoid drug residues in animal products and the possible environmental consequences [21] are also increasingly considered. There is thus a strong impetus for research on alternative approaches to AH drugs [14]. Among those options, evidence has accumulated over the last 20 years to suggest that some bioactive tannin-rich (TR) plants have anthelmintic effects [12, 19]. Legume forages of the Fabaceae family, i.e.: sulla (*Hedysarum coronarium*) [25, 26], big trefoil (*Lotus pedunculatus*) or birdsfoot trefoil (*Lotus corniculatus*) [9, 25, 26], Sericea lespedeza (*Lespedeza cuneata*) [35, 39, 40], or sainfoin (*Onobrychis viciifolia*) [9, 10, 20] have been widely explored. Several studies so far have supported the hypothesis that condensed tannins (CTs) [22, 23] and/or other flavonoids [3, 5] play a significant role in the AH effects of these forages when consumed by animals.

These different data illustrate the concept of nutraceuticals with AH properties [1]. It is generally assumed that the consumption of such forages at an appropriate threshold level of CTs reduces adult worm numbers in the animal through a direct action on the worm [17, 35]. However, this does not provoke their complete elimination *per se*. CTs when in contact with GI nematodes cause modulation of infection dynamics by affecting the biology of different key parasitic stages in the life cycle [11], namely egg excretion, L3 establishment by inhibiting or delaying exsheathment and/or tissue association/penetration supported *in vivo* by a decrease in larvae establishment [6, 17, 30] and possible reduced fertility of worms. The challenge of incorporating such plants in small ruminant production systems would be that when provided as animal feed, the animals will voluntarily eat them in sufficient quantities over time (at least 7 days) in order to successfully affect gastrointestinal parasite biology and ensure beneficial effects on health [13].

In Mediterranean areas, the consumption of local plants composing the rangelands traditionally represents a complementary food resource for livestock husbandry, where and when environmental conditions (e.g. drought) impose feed limits [7, 31]. The communities of plants within the Mediterranean rangelands have moderate to high contents of plant secondary metabolites (PSMs), including tannins [31]. In 2010, an *in vivo* study showed that several of these Mediterranean plants had bioactive properties and could potentially be used as nutraceuticals against GINs in small ruminants [19]. The list included *Pistacia lentiscus*, *Quercus coccifera*, *Castanea sativa* leaves, *Ceratonia siliqua* leaves and pods and sainfoin. The current study will focus on the possible use of a variety of nutraceuticals exploitable within the Mediterranean basin/area.

Sainfoin is a tannin-rich legume forage which can be found in the southern part of Europe and which has been the subject of renewed interest because of several beneficial properties in the context of agroecology. These include AH properties making sainfoin a model of legume forage used as nutraceuticals. Several *in vitro* studies have shown that sainfoin extracts have an effect against different GIN species in a dose-dependent manner [4, 19, 27]. *In vivo* AH effects have also been demonstrated on sheep and/or goats consuming sainfoin by showing a reduction in parasitic egg excretion related to a decrease of female worm fertility and/or of worm numbers depending on the nematode species [30, 41].

Following the same concept and in the area of research on forages, the potential of agro-industrial by-products to be used as nutraceuticals has also been explored. Amongst the potential candidates, carob pods have already drawn some attention since their nutritive value in sheep and goat nutrition has been demonstrated in several studies [32, 43]. In particular, as regards AH properties, Manolaraki et al. [19] obtained preliminary results from *in vitro* and *in vivo* studies in which significant decreases in egg excretion and worm burdens of *H. contortus* and *T. colubriformis* were observed in lambs fed with carob pod meal. Such results suggesting combined nutritive values and potential AH activity in carob pods indicate that this resource might represent a promising model of nutraceuticals derived from agro-industrial by-products.

As mentioned above, the comparison of the respective values of sainfoin vs. carob pods to disrupt the biology of parasitic nematodes relied on a single study in which the animals were experimentally infected with a single challenge of GINs, 2 weeks after the start of the specific diets [19]. Here, our general objective was to confirm previous results of this approach and to be closer to natural conditions of GIN infection, and thus exploring the effects of tannin-rich resources in a model of GIN trickle infections. The specific aims of the present study were: (1) to compare the antiparasitic activity of sainfoin hay and carob pods given as part of a concentrate meal under *in vivo* conditions in lambs which were experimentally trickle infected with two GIN species, (2) to confirm the benefits of these plants on host resilience by measuring certain production and pathophysiological parameters.

## 2. Materials and methods

### 2.1. Trial site

The trial was performed in Greece and precisely on the island of Crete where carob trees are indigenous and carob pod meal is commonly used as animal feed. The facilities hosting the experiment belonged to the Asomati Research Station of HAO Demeter involving animals of the experimental flock. The trial was performed according to welfare rules applied in Greece and its design was approved by the Institute’s ethics committee.

### 2.2. Animals

Sixty-four (32 males and 32 females), 6-month-old, naïve lambs, of the most common local “Sfakion” breed (mean weight: 12.63 ± 0.2816 kg) were used in the trial. The animals were raised indoors under helminth-free conditions. In addition, 10 days before the start of the trial, they were drenched with albendazole at the commercially recommended dose (15 mg/kg). The effect of the treatment was recorded by coprological examination at the start of the study. No anthelmintic resistance was previously reordered in the specific location.

### 2.3. Feeding regimes and experimental design

Two tannin-containing plants were offered to the respective animal groups, the forage legume sainfoin provided as hay *ad libitum* and a meal supplement composed of carob pods. Carob pods were previously dried and crushed to obtain a sort of flour, incorporated in the feed supplement at a concentration of 11% which was the maximum possible (poor energy and protein contents). The free tannin forage lucerne (*Medicago sativa*) hay was used as a control feed for the two extra control groups depending on whether they were drenched with albendazole (group T = negative control) or remained untreated (group NT = positive control) (Table 1). Group T mainly aimed at comparing the effects on resilience between the different infected groups. Refusals were recorded daily. Animals had free access to fresh water. In all the cases, the feed regimes were made isoenergetic and isoproteic and balanced for crude fibre, Ca and P.

**Table 1.** Experimental design: animal diet regimes and/or anthelmintic treatment received according to the four different experimental groups (Total lambs *n* = 64).

Groups	Treatment	Animals
Positive control (NT)	Lucerne hay (*Medicago sativa*)	8 females + 8 males
Negative treated control (T)	Lucerne hay (*Medicago sativa*) + Albendazole drench	8 females + 8 males
Carob (CAR)	Carob pods (crushed) (*Ceratonia siliqua*)	8 females + 8 males
Sainfoin (S)	Sainfoin hay (*Onobrychis viciifolia*)	8 females + 8 males

**Figure 1.**
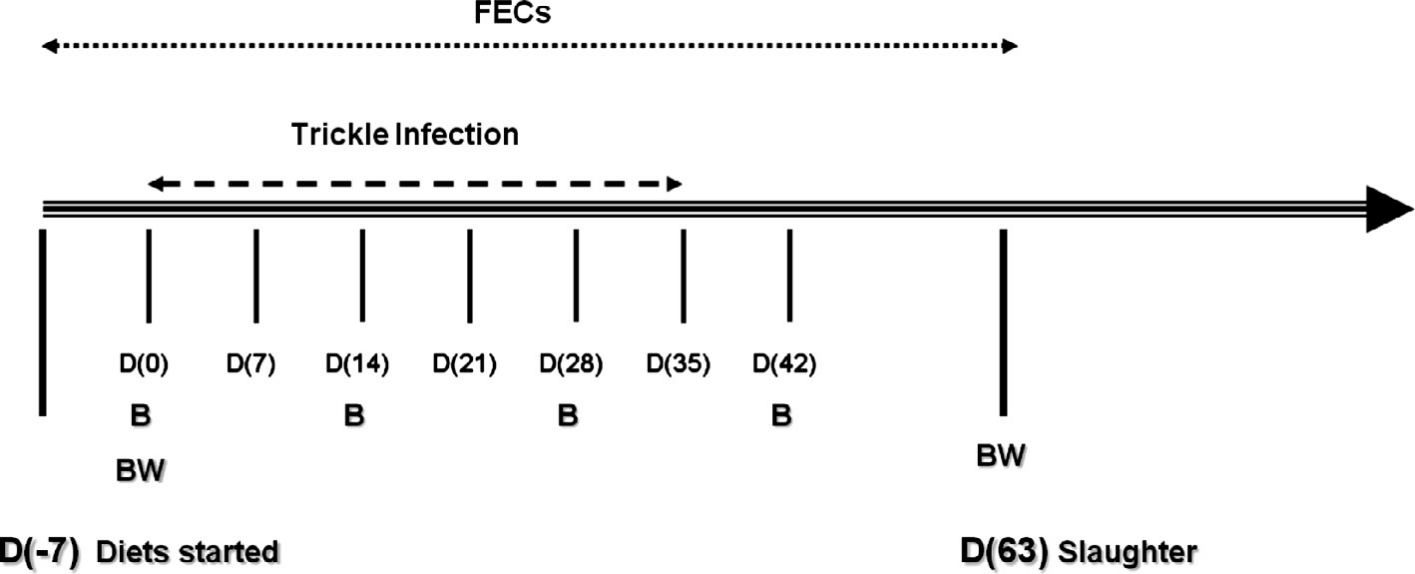
Experimental design: D(-7): animal distribution into feeding regime groups. D(0): start of a weekly trickle infection of *H*, *contortus* and *T. colubriformis* for 6 weeks D(7), D(14), D(21), D(28), D(35) and D(42). D(63): slaughter and necropsy of 8 male lambs par group. Measurements of BW: body weight were performed on D0 and D63, FECs: faecal egg counts (weekly) and B: blood sampling to measure PCV (fortnightly).

Seven days prior to any experimental infection (D-7), the lambs were allocated to the four experimental groups (including 8 female and 8 male lambs per group). The animals were then kept separately per group and the different feeding regimes were applied for 70 days (from D-7 to D63) (Fig. 1). On Day 0 (D0), all animals were experimentally infected *per os* with a mixture of infective third-stage (L3) larvae *of H. contortus* (1000 L3) and *T. colubriformis* (700 L3) given weekly for six consecutive weeks to mimic a natural infection rate [35]. Infective (L3) larvae were cultured from the faeces of monospecifically infected donor lambs and were kept at optimal conditions until infection (4 °C). The age of the larvae at D0 was approximately 2 months in order to ensure infectivity [18].

### 2.4. Parasitological and pathophysiological measurements

Individual measurements were recorded to characterize the effects of the feed on worm biology by evaluating parasitological measurements either *in vivo* (faecal egg excretion) or during the *post mortem* procedures (worm counts and female worm fertility). In addition, measurements were performed to estimate host resilience through production measurements (Body Weight Gain rate [BWG]) by recording the weight at the beginning (D0) and at the end (D63) of the trial and pathophysiological measurements: (Packed Cell Volume [PCV] and serum inorganic phosphate values).

Individual faecal samples were collected weekly directly from the rectum, from D(-7) when the adaptation of diet started, until the end of the assay (D63) to determine faecal egg counts (FECs) according to a modified McMaster technique [33]. Data were expressed as eggs per gram of faeces (EPG).

Individual blood samples were taken fortnightly (D0, D14, D28 and D42) during the trial. Blood was taken by venipuncture of the jugular vein and collected in heparin and heparin-free tubes. The first tubes were used to measure Packed Cell Volume (PCV) according to the microhaematocrit method. Serum was collected from heparin-free tubes, separated and frozen to determine inorganic phosphate values using a photometric assay [34].

On D63 (end of the assay), only lamb males were slaughtered (8 animals per group) and the female lambs were drenched with albendazole at the commercial recommended dose (15 mg/Kg) and returned to the flock. After necropsy, total adult worm burdens and fertility of female worms for the two nematode species (*H. contortus* and *T. colubriformis*) were evaluated from the abomasum and small intestine [8]. At necropsy, the gastrointestinal tracts were immediately removed and the abomasa and small intestines were gently washed to collect the luminal contents. Moreover, the abomasal mucosa were digested using a pepsin digestion procedure, after 4 h of incubation in a pepsin solution at 37 °C in order to obtain the larval stages.

Worm counts were performed according to a 10% aliquot technique. Identification of worm stages, sex and species were conducted using a standard procedure [18]. Thereafter, the fertility of the female worms was evaluated from 10 females per lamb of *H. contortus* and/or *T colubriformis*. The fertility of *T. colubriformis* was estimated by the direct counting of eggs *in utero* after clearance with lactophenol. For *H. contortus*, the fertility was determined according to the method described by Kloosterman et al. [16].

### 2.5. Statistical analysis

Statistical analyses were performed to compare the values for BWG and pathophysiological measurements in the four experimental groups (using both T and NT controls as positive and negative indicators). For the parasitological parameters (namely EPG, worm counts and female worm fertility), the statistical analyses were restricted to the three infected groups (namely NT, CAR and S groups). The group treated with AH (T group), although FECs were also recorded in the same time intervals to ensure efficacy, was excluded from this analysis. In addition, the values of FECs, worm burden and fertility of females data were log_10_ (X + 1) transformed before statistical analysis in order to normalize the distribution. Lastly, the analyses of worm fertility were restricted to *H. contortus* because of the patchy distribution and insufficient number of *T. colubriformis* worms in many lambs of the different experimental groups. All analyses were performed by using SYSSTAT 9.0 Software. AUC values for the EPGs of each animal were calculated using Graphpad Prism software for Windows (version 5.01). AUC values were compared by using one-way analysis of variance (ANOVA) and *post hoc* comparisons with Bonferroni adjustment.

For the worm counts (*H. contortus*, *T. colubriformis* and total number [total of both nematode species]), the differences in worm burden mean number and fertility were examined by using ANOVA after log (WN + 1) transformation of the data. Similarly, an ANOVA 2 was applied to examine the differences in egg count per female *H. contortus* worms taking into account the treatment and the individual animal.

To compare the differences in body weight gains, ANOVA was performed on the BW rates calculated between D-7 and D63. For log transformed FECs, PCV and inorganic phosphate values, the statistical comparisons were first performed by use of repeated measures ANOVA completed by a date-to-date one-way analysis of variance (ANOVA), including *post hoc* tests with Bonferroni correction. For PCV, because of initial differences on D0, the date-by-date analysis was performed by using the D0 values as covariate.

## 3. Results

### 3.1. Parasitological parameters

#### 3.1.1. Egg excretion

**Figure 2.**
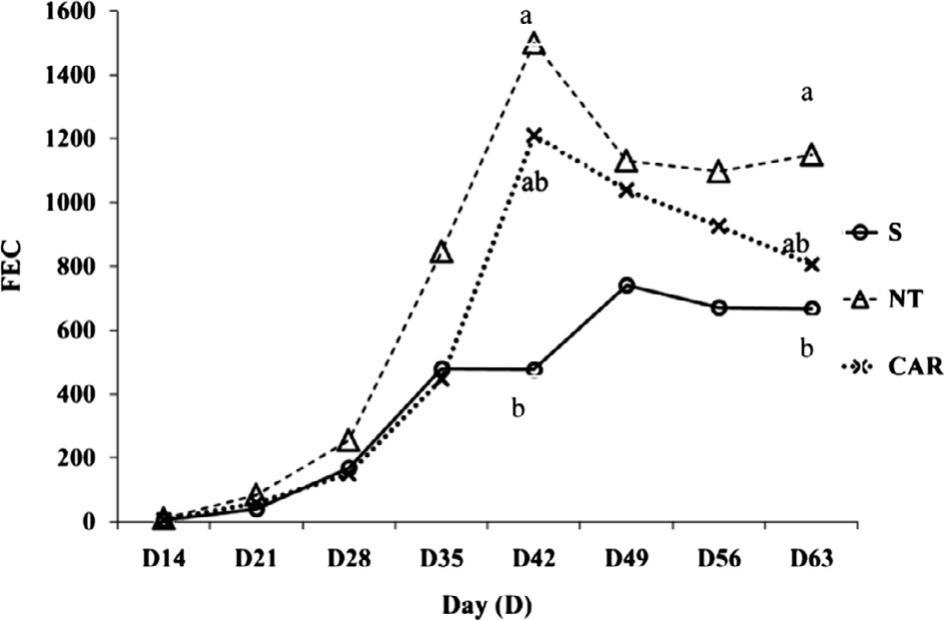
Faecal egg counts (arithmetic mean of eggs per gram of faeces) based on the different diet regimes over the study period (D14–D63). S (sainfoin), NT (non-treated/positive control), CAR (carob).

Analyses of the egg excretion values based on the Analysis of Variance on Repeated Measures have shown overall differences close to significance (*P* < 0.06) between the CAR, S and NT groups (Fig. 2). The values of FEC in the sainfoin were regularly lower compared to those of the NT group, followed by the values of lambs receiving carob. Results of the date-by-date analyses were significant only on D42 and D63 (*P* < 0.05) with the values in the sainfoin group differing from those in the NT group. On these two dates, the percentage of egg reduction was measured using the following formula: %Reduction = ((Total amount C − Total amount Treated)/Total amount Control) × 100. Compared to the NT (positive control) groups fed with lucerne (*Medicago sativa*), the results showed a reduction of −68% for the S group and of −19% for the CAR group on D42. On D63, these reductions in EPGs were, respectively, −42% and −30% for the S and the CAR groups.

The mean AUC value (indicator of total number of eggs excreted) for S group (29,662.5, SD ± 15,103.3) was reduced by 46.93% compared to the NT group (38,456.25, SD ± 27,873.2) which was significantly different (*p* = 0.034). As regards the CAR group, although the mean AUC value (20,405, SD ± 10,764.72) was reduced by 22.86% compared to the NT group, this reduction was not significantly different (*p* = 0.614).

As regards group T, the mean FECs throughout the study were 1.25, 0, 0, 6.25, 10, 17.5, 16.25 and 37.5 for sampling dated D14 to D63, respectively.

#### 3.1.2. Worm counts

**Figure 3.**
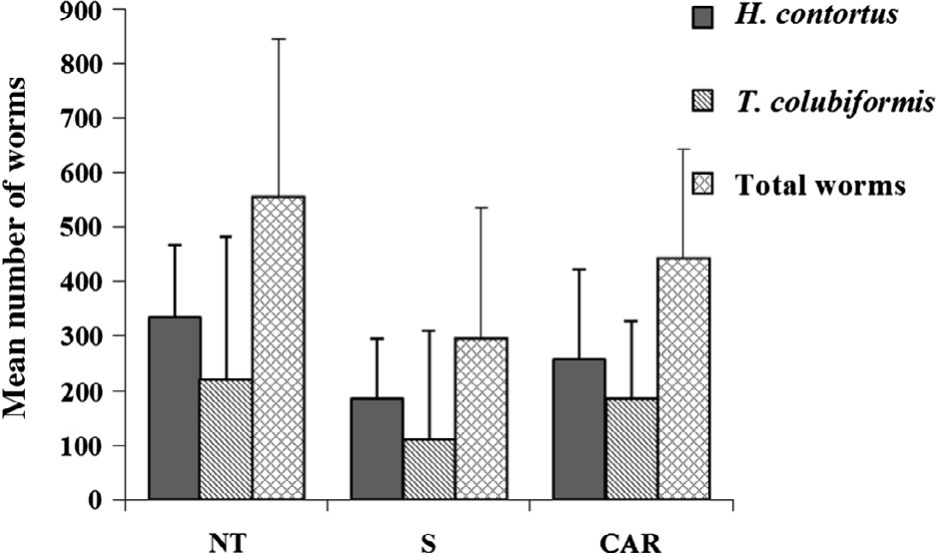
Mean number of worms of the two nematode species recovered and of the total worm number in the different experimental groups. S (sainfoin), NT (non-treated/positive control), CAR (carob).

The total mean worm burdens (Fig. 3) for *H. contortus* (Hc) and *T. colubriformis* (Tc), found at necropsy per group (8 males), were respectively: 72 ± 87 (Hc: 26 ± 44; Tc: 46 ± 88) in group T; 295 ± 241 (Hc: 185 ± 109; Tc: 110 ± 200) in group S; 442 ± 201 (Hc: 257 ± 164; Tc: 185 ± 140) in group CAR, and 556 ± 289 (Hc: 335 ± 131; Tc: 221 ± 259) in group NT. The number of worms in the treated group (T) differed significantly from the three others. A trend (*p* < 0.11) was found for a reduced total worm number in the S group compared to the NT controls. No significant differences were found in the number of *H. contortus* between the three treated groups, but a significant reduction (*p* < 0.05) was measured in the number of *T. colubriformis* between the S vs. the NT groups. When compared to the NT control groups, the percentages of reductions were respectively −45% for *H. contortus* and −50% for *T. colubriformis*, in the S group and only −23% and −16% for the CAR group (Fig. 3).

#### 3.1.3. Fertility of female worms

The mean values of female *H. contortus* fertility were respectively 91 eggs per female in the control NT group, 75 in the S group, and 67 in the CAR group. These differences were non-significant.

Because the mean number of *T. colubriformis* was low and highly variable between animals per group it was not possible to evaluate any statistical difference in the female fertility for this intestinal species.

### 3.2. Production measurements

Overall, the animals were well adapted to the feed offered and no refusals were recorded until the end of the study.

The BWG calculated over the 70-day experimental period was respectively 4.16 (SD ± 0.89) kg for group NT, 4.72 (SD ± 0.77) kg for group C, 4.69 (SD ± 0.81) kg for group CAR and 4.81 (SD ± 1.01) kg for group S. No statistical differences between groups were found depending on their diet for the BWGs, comparing initial and final BW data, although S group showed a 13.7% higher BWG than animals in the control group. Nonsignificance was documented when the final BWs of the treated groups were compared to the NT control group.

### 3.3. Pathophysiological parameters

The analysis of variance on repeated measurements for PCV values (from D14 to D56) revealed overall statistical differences between the groups (*p* < 0.001; Table 2). In addition, when the date-by-date ANOVA 1 was applied, using the D0 values as covariate, statistical differences (*p* < 0.05) were found between groups on each date (*p* < 0.01). In contrast to the control negative (T) group, which showed repeatedly the highest PCV values, the values in S group were found to be statistically different only on D28. All values and statistical differences found in different groups are presented in Table 2.

**Table 2.** Packed Cell Volume values (PCV) (normal style) and serum inorganic phosphate values (*italics*) on the different specific days of the trial (±SD). NT = positive control, T = treated, negative control, S = sainfoin fed group; CAR = Carob fed group. Different superscripts in the same column indicate significant differences.

Group/day	D0	D14	D28	D42	D56
(NT) Untreated control+	35.4^ab^ ± 4.6 *6.8 ± 1.4*	30.0^b^ ± 3.2 *7.4 ± 1.3*	29.4^c^ ± 3.7 *6.8 ± 0.8*	32.8^b^ ± 4.5 *10.0*^*a*^ *± 2.0*	28.4^b^ ± 2.1 *8.0 ± 1.4*
(T) Treated control−	38.7^ab^ ± 6.0 *5.2 ± 1.2*	38.6^a^ ± 4.2 *8.3 ± 1.8*	38.1^a^ ± 4.3 *6.1 ± 1.7*	41.9^a^ ± 4.3 *8.5*^*b*^ *± 1.8b*	33.6^a^ ± 4.1 *8.2 ± 2.5*
(CAR) carob	34.8^b^ ± 2.8 *7.1 ± 1.4*	30.6^b^ ± 3.9 *8.1 ± 1.5*	29.6 ± 4.2 *7.0 ± 2.4*	33.4 ± 2.5 *8.2*^*b*^ *± 1.6*	29.6^b^ ± 2.4 *7.6 ± 1.1*
(S) sainfoin	43.2^a^ ± 6.8 *5.7 ± 1.8*	34.6^a^ ± 5.0 *8.9 ± 1.7*	32.8^b^ ± 5.1 *6.7 ± 1.4*	37.9^a^ ± 4.0 *7.6*^*b*^ *± 1.2*	32.5^a^ ± 3.9 *7.0 ± 0.9*

Unlike for PCV, the inorganic phosphate serum concentration records showed wide variability in values and few statistical differences between groups except on D42, when significant differences were found between the NT lambs and the three other groups (*p* < 0.01) (Table 2).

## 4. Discussion

The general objective of our study was to compare the effects of feed based on carob pods and sainfoin hay on the worm populations and the host resilience of infected lambs. This comparison aimed at evaluating the potential exploitation as nutraceuticals of two types of local TR resources (a legume forage vs. an agro-industrial by-product) in the same conditions of ruminant breeding and GIN infections. This aims at complementing results of a previous study [19] that was performed with similar objectives. However, this first study was performed based on a single initial infection with both GIN species. In this study, we aimed at confirming the previous results under conditions which better mimic the natural conditions of infection in order to prepare future implementation under farm conditions. One of the interests of the comparison is due to the fact that we used the same tannin-rich resources in both studies.

Egg excretion (EPG) was the main parasitological measurement performed *in vivo* in the different groups. The results showed some regular decreases in the mean values of EPG in the CAR and S groups compared to the NT group. However, statistical differences were only recorded between the S and NT groups. These results with sainfoin corroborate previous ones illustrating that one of the main effects in lambs or kids related to the consumption of a range of tannin-rich legume forages was a decrease in nematode egg output [10, 24, 41, 42]. Previous results on the effects of carob pods in GIN infected small ruminants are less abundant than with sainfoin. In the previous study [19], significant decreases in FEC were also measured in the lambs fed on carob pods. Here, although some reductions in egg output were measured (based on direct analyses of the egg excretion and the area under the curve), the differences to the control groups were non-significant.

We aimed at analysing the origin of these decreases in egg excretion based on necropsy data. They concerned both worm numbers and female worm fertility for the two GIN species as performed in previous studies [19, 29]. Here, the decreases in EPG in the S group compared to the NT controls were associated with a trend for a decreased total worm burden which was mainly related to a significant reduction in the number of intestinal worms but with no effect in the number of *H. contortus*. In contrast, no significant differences were observed in worm numbers or in worm fertility in the carob group. This is consistent with the lack of significant differences in EPG in the CAR vs. the NT groups.

A higher AH effectiveness of some tannin-containing plants against intestinal worm species, such as *T. colubriformis*, has already been found for sainfoin in goats [29] and lambs. Higher susceptibility of intestinal species compared to abomasal ones to the effects of tannin-rich resources has also been described with quebracho [2]. However, some other reports mentioned a higher susceptibility to TR resources for abomasal species when compared to intestinal ones [12]. Such contradictory results on plant-AH effects between abomasal vs. intestinal GIN species and/or location need to be further studied.

In the previous research results [19], significant reductions were measured in the female fertility of *T. colubriformis* in lambs fed with carob and on both *T. colubriformis* and *H. contortus* in lambs receiving sainfoin. The current results indicated in the case of *H. contortus*, fertility values which were lower by 25.8% and 18% for the *C. siliqua* and *O. viciifolia* fed regime, respectively, when compared to the worms collected in the NT control lambs fed on *M. sativa*. However, these differences were non-significant. It is worth noting that, when compared to the values observed in the first study, the mean number of eggs per female *H. contortus* was low, even in the control group, and the low and highly variable number of worms. Unfortunately, the heterogeneous prevalence of *T. colubriformis* in the lambs did not allow any statistical comparisons on the fertility of the female *T. colubriformis.*

It is suspected that such diversity in results (effects on both worm numbers and/or female worm fertility) between the two studies on carob pods and sainfoin hay might be explained to some degree by (i) differences in the infection regimes (single vs. trickle) which involved different nematode stages being subjected to the direct effects of tannin-containing resources, or (ii) by possible consequences on immune host response. Clearly, on the one hand, a single infection combined with the consumption of TR resources targeted the established adult worm populations [19], and on the other, a mode of trickle infections, as in the current study, exposed different parasite life stages (from L3 to adult worms) to the action of tannin-containing resources. In addition, it is suspected that the trickle mode of infection, which was applied in the current study, was better designed to stimulate local host immune responses [13, 42] which are less favoured in case of the single mode of infection.

Evidence obtained from *in vitro* assays with the addition of PVPP (an inhibitor of polyphenols, namely tannins and flavonoids) suggests a key role for these plant secondary metabolites in the AH properties for both sainfoin and carob waste pods [19]. Subsequently, different effects on biological functions or traits of different parasitic stages could be expected [12].

Contradictory results have been described about the effects of carob on body host performance and physiology. Recent results revealed no differences in body performances, voluntary intake and body gain rate for adult lambs fed with carob pods, in contrast to control groups [28]. This contrasts with previous results where body performance was compromised, by carob pulp feeding [32] due to the possible adverse effects of CTs in carob pods [36, 37]. These differences may depend on qualitative and/or quantitative differences of carob fruits (example: pulp, pod or flour) [28]. In our study, carob pods comprising the pods were used to obtain a powder and no differences were found in body gain rate between groups, although the sainfoin groups showed a 13.7% increase in live weight gain compared to the control ones. This last result confirmed previous ones obtained by comparing sainfoin and lucerne groups in ruminants [44].

Two pathophysiological measurements were performed to evaluate the impact of carob pods and sainfoin hay on host resilience, in order to withstand the negative effects of GINs. They were respectively related to *Haemonchus* (PCV) and *Trichostrongylus* (serum inorganic phosphate) infections. Statistical differences in the mean PCV values were found throughout the assay with, overall, higher values in the S and T groups compared to the CAR and NT lambs. These results with sainfoin tend to confirm previous data suggesting positive effects of the consumption of tannin-containing legume forages on the resilience of animals [25, 29]. In contrast to PCV, the analyses of inorganic phosphate levels showed high variability in values and the comparisons between groups were not consistent. Reductions in seric phosphate levels have been associated with worm infection of the small intestine, particularly with *Trichostrongylus* sp. [29]. The decreases in phosphate absorption have been related to the damage induced to the intestinal mucosae (abrasion of villi, alteration of epithelial cells and of their equipment in digestive enzymes). The low level of mean infection and the variability of infection observed with *T. colubriformis* between individual lambs are probably two main reasons explaining the lack of any trend observed between experimental groups in the phosphate values observed in the current study.

In summary, results of the current study (1) tend to confirm an effect after administration of both tannin-rich resources (although stronger for sainfoin) on egg excretion and subsequent pasture contamination as a likely outcome; (2) demonstrate that significant effects were obtained in the sainfoin group on worm numbers particularly on *T. colubriformis* (*p* < 0.05). This might be explained by reduced L3 establishment in early parasitic stages; (3) reveal that the AH effects were less consistent (and usually non-significant) with carob, potentially due to the limitations of its concentration in the ratio (high level of sugar but low proteins and lipids).

## 5. Conclusions

The importance of the use of local forages, plant parts or plant extracts in helminth control relies on their sustainable and environmental qualities [38]. Experience on the antiparasitic effect of *Ceratonia siliqua* remains rare. Due to the difficulties and the amount of variables that could interfere with pathophysiological, parasitological and body performance results, further investigations are needed to confirm the AH effects hypothesis and the type of secondary metabolites involved in the use of carob as a nutraceutical.
